# Time- and Kellgren–Lawrence Grade-Dependent Changes in Intra-Articularly Transplanted Stromal Vascular Fraction in Osteoarthritic Patients

**DOI:** 10.3390/cells8040308

**Published:** 2019-04-03

**Authors:** Tung Dang Xuan Tran, Chi-Ming Wu, Navneet Kumar Dubey, Yue-Hua Deng, Chun-Wei Su, Tu Thanh Pham, Phuong Bich Thi Le, Piero Sestili, Win-Ping Deng

**Affiliations:** 1 School of Dentistry, Taipei Medical University, Taipei 11031, Taiwan; d204105004@tmu.edu.tw; 2 Van Hanh Stem Cells Unit, Van Hanh Hospital, Ho Chi Minh City 700000, Vietnam; thanhtuvanhanh@gmail.com; 3 Graduate Institute of Biomedical Materials and Tissue Engineering, College of Biomedical Engineering, Taipei Medical University, Taipei 11031, Taiwan; chiming.wu@jade-dental.com.tw (C.-M.W.); bioengineer.nkd@gmail.com (N.K.D.); 4 Stem Cell Research Center, College of Oral Medicine, Taipei Medical University, Taipei 11031, Taiwan; q7s5w8a4@gmail.com; 5 Department of Life Science, Fu Jen Catholic University, New Taipei City 242, Taiwan; yuehuahua828@gmail.com; 6 Department of Pulmonary Medicine, Vietnam Military Medical Academy, Ha Noi 12108, Vietnam; drbphuong@gmail.com; 7 Dipartimento di Scienze Biomolecolari, Università degli Studi di Urbino Carlo Bo Via “I Maggetti” 26, 61029 Urbino, Italy; piero.sestili@uniurb.it

**Keywords:** knee osteoarthritis (OA), KL grade, stromal vascular fraction (SVF), MRI, WOMAC, VAS, OS, BME

## Abstract

Knee osteoarthritis (OA) is one of the most prevalent disorders in elderly population. Among various therapeutic alternatives, we employed stromal vascular fraction (SVF), a heterogeneous cell population, to regenerate damaged knee cartilage. OA patients were classified on the basis of age, gender, body mass index (BMI), and x-ray-derived Kellgren–Lawrence (KL) grade. They were treated with SVF and followed-up for 24 months. Visual analogue scale (VAS) and Western Ontario and McMaster Universities Osteoarthritis (WOMAC) Index were used to determine treatment efficacy. Cartilage healing was assessed using the MRI-based Outerbridge score (OS) and evaluation of bone marrow edema (BME) lesions, while a placebo group was used as a control. Time- and KL-dependent changes were also monitored. We observed a decreasing trend in VAS score and WOMAC index in the SVF-treated group up to 24 months, as compared with the placebo group. Besides, a significant increase and decrease in Lysholm and OS, respectively, were observed in the treatment group. Compared with the values before treatment, the greatly reduced WOMAC scores of KL3 than KL2 groups at 24 months, indicate more improvement in the KL3 group. Highly decreased BME in the treated group was also noted. In conclusion, the SVF therapy is effective in the recovery of OA patients of KL3 grade in 24 months.

## 1. Introduction

Knee osteoarthritis (OA) is one of the most common progressive joint disorders, especially among elderly population in the United States and other developed countries [1,2,3]. Cartilage devolution, stiffness, loss of joint function, bone loss/rearrangement, and pain are primary characteristics of OA [4,5]. In the clinics, OA patients are categorized on the basis of their Kellgren–Lawrence (KL) grades (1 to 4), whose range of symptomatic characteristics includes the narrowing of the joint space to definite deformity of bone ends [6]. Multiple risk factors for OA include age, gender, inflammation, genetics, mechanical wear and tear during exercise, sports, or any other stressful activity [7,8,9,10]. There is wide perception that obesity and increase in life expectancy are major causes of the increase in OA in the last decades; however, a recent study carried out by Wallace et al. suggests that life longevity and body mass index (BMI) are not the only factors for the increase in OA, and extensive research is needed to determine other factors associated with OA increase [11]. The self-renewal ability of chondrocytes is significantly lost in aged persons (>60 years), and this severely affects cartilage structure and maintainance [12]. Moreover, it has also been established that the secretion of proteolytic enzymes such as aggrecanases and metalloproteinases further degrades the damaged cartilage [13,14]. OA-related pain is treated by non-pharmacological approaches such as physical therapy, yoga, land- and water-based exercise, tai chi, and weight loss [15,16,17,18,19,20], as well as with pharmacological agents such as nonsteroidal anti-inflammatory drugs (NSAIDs) [21,22], chondroprotective compounds, calcium, opioids [23,24], and hormones [25]. Hyaluronic acid (HA) is intra-articularly administered to restore the viscoelastic properties of injured cartilage [26,27]. Surgical treatments including arthroscopy, microfracture, subchondral drilling, and abrasion arthroplasty are used to treat late-stage OA; however, the limitations of these procedures include the formation of fibrocartilage, which has less ability to absorb shock, thereby compromising the functional characteristics of the native cartilage tissues [25].

An alternative surgical technique, the autologous chondrocyte implantation (ACI), has been recently used to overcome the limitations associated with the previously mentioned surgical techniques. ACI is a common surgical intervention to promote healing of cartilage injuries in OA [28,29]. However, the effectiveness of ACI is restricted because of the limited availability of chondrocytes and the compatibility between implanted chondrocytes and host site [30]. Cell-based regenerative therapies along with biomaterials, especially stem cells and hydrogels, are emerging and promising procedures to counter OA. Bone marrow-derived stem cells (BM-MSCs), peripheral blood-derived stem cells, adipose-derived stem cells (ADSCs), and synovial fluid-derived stem cells have been studied in the presence or absence of biomaterials [31]. The paracrine effects of stem cells have been widely associated with regeneration and repair activities [32]. The adipose tissue is considered a rich and preferable source of stem cells due to the feasibility of harvesting tissue and isolating stem cells. 

Stromal vascular fraction (SVF) is a heterogeneous population of various immune, precursor, progenitor, and stem cells. SVF is considered to be as equal as or sometimes more effective than ADSCs; therefore, it provides other functional advantages, such as structural support, over ADSCs [33,34,35,36]. However, SVF is immunologically restricted because of the presence of various cells and only fit for autologous treatment [37], whereas, ADSCs are multipotent cells that can differentiate into chondrocytes, with capability of self-renewal, high plasticity, and immunomodulatory and anti-inflammatory properties [38,39]. SVF has been widely studied as an alternative therapeutic agent to treat sclerosis, myocardial and bone-related disorders, blood vessel regeneration, and pulmonary diseases [40,41,42]. Recent works have also been extensively focused on evaluating SVF potential in orthopedic ailments [41,42]. Various clinical studies combining SVF with  plasma-rich protein (PRP) , hyaluronic acid (HA), ceramic and fibrin glue were carried out to assess the potential of SVF in the treatment of OA [43,44,45]. Considering the therapeutic significance of SVF, this study was carried out to assess the therapeutic efficacy of SVF in OA treatment through the regeneration of articular cartilage. During our study, we specifically investigated time- and KL grade-dependent changes up to 24 months.

## 2. Materials and Methods

### 2.1. Study Design and Participants

This study was an open-label, single-center, non-randomized, placebo-controlled, phase I/II clinical trial to evaluate the improvement in knee pain and knee function, as well as cartilage restoration. The 33 patients enrolled in the study were deliberately allocated to two groups, which were designated arthroscopic microfracture treatment only and arthroscopic microfracture treatment combined with SVF injection. Observation and follow-up data were recorded after 12 and 24 months. The eligibility criteria included: osteoarthritic knee joint with KL grades 2–3 and age >38 years. Patients meeting the following criteria were excluded: autoimmune or inflammatory disease, infection requiring parenteral administration of antibiotics, serious internal disorders, corticosteroids or viscosupplements injection into the affected knee within the past 3 months, and stiffness due to previous severe injury. The protocol was approved by the Viet Nam Ministry of Health (No. 2288/QDBYT) and the Ethical Committee in Biomedical Research of Van Hanh General Hospital (No. 90-084/QD-BVVH). Patients participating in this research provided an informed consent, in accordance with the Declaration of Helsinki.

### 2.2. Fat Tissue Harvest and SVF Isolation

Lipoaspirates were harvested from patients’ lower abdomen by a standard liposuction technique. Briefly, through incision, a solution of tumescent lidocain, 250 mL of normal saline, 0.9% and 0.2 mL of 1:1000 epinephrine was injected in the subcutaneous fat. Thereafter, 50–100 mL of lipoaspirate was collected through Triport Harvester Cannula (Tulip Medical Product, CA 92117 USA), and a 60 mL Luer-lock syringe. The SVF from the lipoaspirate was isolated by means of collagenase digestion (Collagenase NB 6 GMP Grade, Nordmark Biochemicals, Ho Chi Minh City, Vietnam) and the ADSC Extraction Kit (Geneworld Co. Ltd., Ho Chi Minh City, Vietnam) approved by the Viet Nam Ministry of Health. The SVF was then washed thrice with sterile PBS to remove collagenase. Finally, the SVF was diluted with normal saline 0.9% to obtain 6 mL of solution containing 90–120 million cells to administer in each knee joint.

### 2.3. Arthroscopy Microfracture Procedure

Spinal anesthesia for knee arthroscopy was done by using 2 mL (5 mg/mL) bupivacaine hydrochloride. The debris, crystal, and synovitis were removed, and microfracture holes were placed 3–4 mm apart by the arthroscopy microfracture technique, as described by Steadman et al [46]. After arthroscopy, the knee joint was drained for 6 hours, and the drainage tube was withdrawn before the injection of the SVF. The rehabilitation period of the patients under the guidance of a physician included three time points. In the first 6 weeks, walking with crutches, partial weight bearing, and passive motion of the joint up to 90° were allowed. During 6–12 weeks, normal walking in combination with the use of a knee protector and quadriceps and hamstring training were performed. After 12 weeks, balance and core training with unlimited knee joint movement was administered.

### 2.4. Follow-Up and Evaluation

Patients were monitored in the hospital for one week post-arthroscopy. After this, patients were followed for 24 months. Clinical manifestations such as pain, stiffness, and functional mobility were substantially recorded. Western Ontario and McMaster Universities Arthritis Index (WOMAC) [47], Lysholm [48], and visual analogue scale (VAS) scores were assessed before treatment and at 12 and 24 months after surgery. Magnetic resonance imaging (MRI) was performed before treatment and at 12 and 24 months after treatment. Specifically, the MRI analysis was performed to assess the extent of cartilage damage according to the Modified Outerbridge Classification [49].

### 2.5. Statistical Analysis

The data are expressed as the mean ± SD. The comparisons between groups were performed by one-way analysis of variance (ANOVA) and *t*-test, using SPSS-22 (IBM, New York, NY, USA), and *p* values <0.05 were considered statistically significant.

## 3. Results

### 3.1. Patient Characteristics

**Figure 1:**
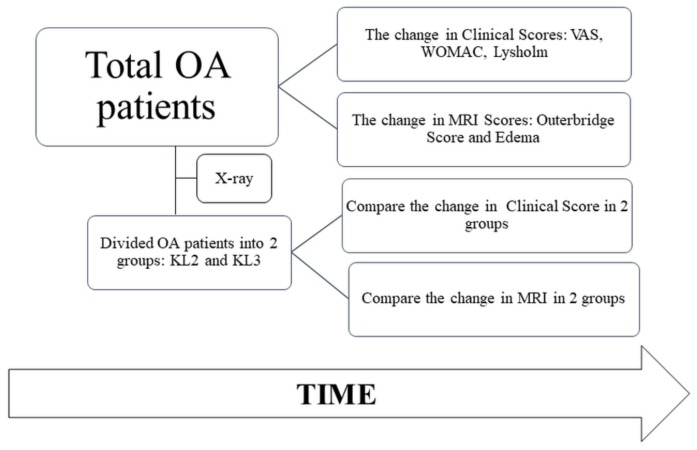
The schematic of the study, which shows that the osteoarthritis (OA) patients were identified on the basis of their clinical and MRI scores, in addition to x-ray-dependent Kellgren–Lawrence (KL) grades. These pateints were further treated with stromal vascular fraction (SVF), and all the outcome scores were assessed after 12 and 24 months.

The study was conducted from September 2014 to June 2017 at Van Hanh Hospital, Ho Chi Minh city, Vietnam. The overall schematic is illustrated in Figure 1, which shows that the OA patients were identified on the basis of their clinical and MRI scores, in addition to x-ray-dependent KL grades.

Eighteen patients who satisfied the exclusive and inclusive criteria were selected to receive the treatment of SVF, a heterogeneous cell population containing mesenchymal progenitor/stem cells, preadipocytes, endothelial cells, pericytes, T cells, and M2 macrophages [50]. The demographic characteristics of the patients are shown in Table 1.

The patients were classified on the basis of their age, gender, BMI, and KL grade (Table 1). In general, the two groups (SVF treatment and placebo) shared quite similar demographic characteristics.

### 3.2. Changes in VAS and Western Ontario and WOMAC Index after SVF Treatment

**Figure 2:**
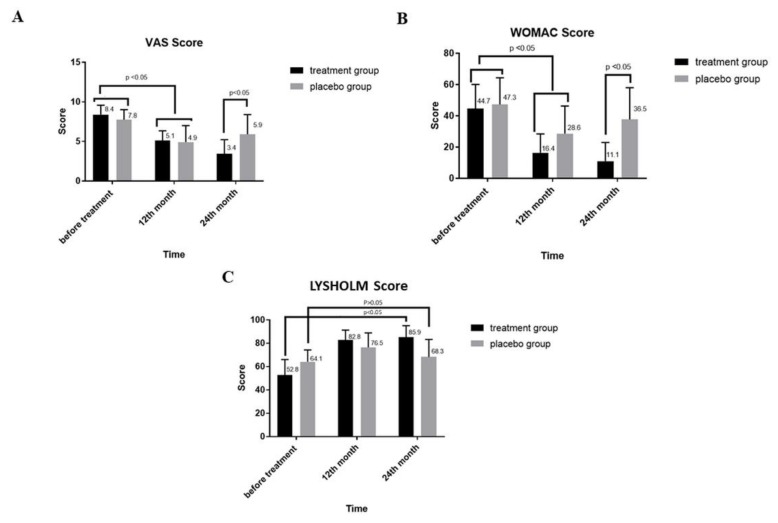
Assessment of clinical outcomes of OA patients treated with SVF at 12 and 24 months. (**A**) Visual analogue scale (VAS) score (**B**) Western Ontario and McMaster Universities Arthritis Index (WOMAC) index, and (**C**) Lysholm score of the SVF-treated group compared to the placebo group.

VAS is a reliable scale for the assessment of pain in osteoarthritic condition [51], whereas WOMAC includes a questionnaire about pain, stiffness, and inability of conducting activities in daily life [52]. In both scales, the lower score represents a better functional status of the patient. The effects of the SVF treatment on the VAS and WOMAC scores of KL2 and KL3 patients are represented in Figure 2A,B, respectively. The results revealed that after 12 months, no significant difference was found between the VAS scores of the SVF treatment and placebo groups (5.1 ± 2.5 vs. 4.9 ± 2.4). However, both scores were significantly decreased compared to that before the SVF treatment (*p* < 0.05). Further, as compared to the placebo group, a sharp decreasing trend in the VAS score of the treatment group was observed up to 24 months. The VAS score in the treated group continuously reduced after 12 and 24 months. Specifically, compared to the mean VAS score at 12 months, the score at 24 months was significantly reduced (5.1 ± 1.2 vs. 3.4 ± 1.8, *p* < 0.05). On the contrary, the score of the placebo group after 12 and 24 months increased from 4.9 ± 2 to 5.9 ± 2.47, but this difference was not significant. A similar trend was also observed for the WOMAC score in the placebo group, which was significantly decreased after 12 months of treatment (47.3 ± 17.1 vs. 28.6 ± 12.7, *p* < 0.05). However, a significant increase was observed thereafter at 24 months (36.5 ± 20.3 vs. 28.6 ± 12.7, *p* < 0.05). Meanwhile, the WOMAC score in the treated group decreased sharply after 12 months (44.7 ± 15.4 vs. 16.4 ± 12.1, *p* < 0.05) and further declined significantly to 11.1 ± 11.9 at 24 months (11.1 ± 11.9 vs. 16.4 ± 12.1, *p* < 0.05). Overall, at 24 months, both VAS and WOMAC scores in the placebo and treatment groups diminished compared with the scores before treatment. However, the decreasing trend in the treatment group was larger than in the placebo group, which is indicative of improvement after SVF therapy.

### 3.3. Changes in Lysholm Score after SVF Treatment

The Lysholm Knee Scale is another recommended measure of knee function [48]. As per Lysholm scale interpretation, a higher score represents better knee function. Before treatment, the Lysholm scores of the placebo and treatment groups showed a significant difference (64.1 ± 10.2, 52.8 ± 13.2; *p* < 0.05) (Figure 2C). The results showed that the score of the placebo group increased to 76.5 ± 12.4 after 12 months; thereafter, a notable decrease was recorded after 24 months (68.3 ± 15.0). However, the overall increase from the value before treatment to that at 24 months in the placebo group was found not to be significant (64.1 ± 10.2 vs. 68.3 ± 15.0). Similarly, the treatment group showed no statistically significant increase in Lysholm score after 24 months, compared to 12 months. However, compared to the value before treatment, this score was significantly increased at 24 months (52.8 ± 13.2 vs. 85.9 ± 9.9, *p* < 0.05), implying an improvement in knee function.

### 3.4. MRI-Based Evaluation of Bone Edema and Cartilage Healing

**Figure 3:**
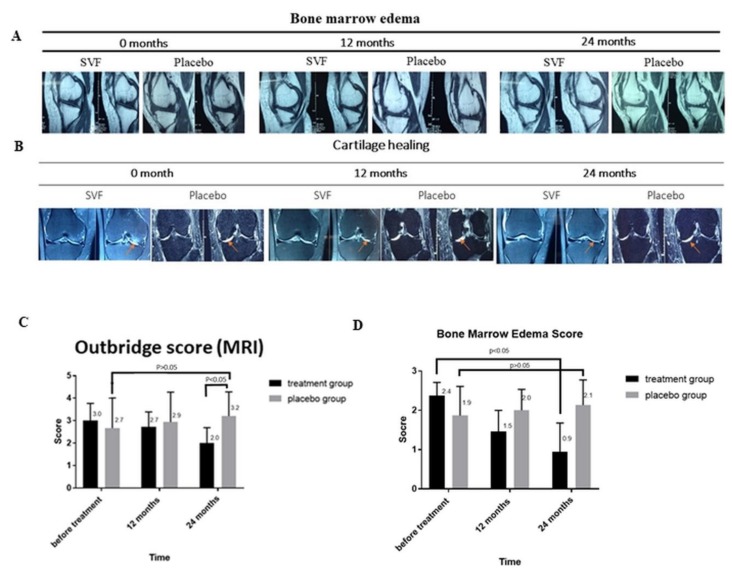
MRI analysis of OA knee-joints after SVF therapy. (**A**) Bone marrow edema (BME) and (**B**) (**B**) Cartilage healing and decrease in bone marrow edema (orange arrow) determined though the Outbridge score (OS) at 0, 12, and 24 month, respectively. (**C**) Cartilage injury evaluation by OS scores indicating the depth of defect in cartilage lesions before treatment and at 12 and 24 months after treatment in placebo and SVF-treated groups. (**D**) Length of BME lesions before and 12 and 24 months after treatment in placebo and treatment groups.

MRI results showed that after 24 months of treatment, bone marrow edema was decreased in both the placebo and the SVF treatment groups; however, the decrease in bone marrow edema in the SVF treatment group was larger (22 mm vs. 8 mm) than in the placebo group (20 mm vs. 12 mm) (Figure 3A). Similarly, the Outbridge score was decreased from 4 (at 0 months) to 3 (at 12 months) and 1 (at 24 months), implying a considerable improvement in cartilage generation in the SVF-treated group (Figure 3B).

### 3.5. Cartilage Injury Evaluation by MRI-based Outerbridge Score

The level of cartilage injury was measured by the Outerbridge score (OS) [53]. The OS of the study groups were recorded on the basis of MRI examination for assessment of cartilage lesions, particularly, depth of defect (Figure 3C) [54]. In the placebo group, the OS score increased slightly after 12 months (2.7 ± 1.3 vs. 2.9 ± 1.3), and this trend was maintained up to 24 months (3.2 ± 1.1). On the contrary, as compared to the values before treatment, the OS score in the treated group decreased after 12 and 24 months from 3.0 ± 0.8 to 2.7 ± 0.7 and 2.0 ± 0.7, respectively. The OS score pattern initially showed no significant difference between placebo and treatment groups (2.7 ± 1.3 vs. 3.0 ± 0.8); however, after 24 months, a significant difference between the OS scores of the two groups could be observed (3.2 ± 1.1 vs. 2.0 ± 0.7; *p* < 0.05). Taken together, the OS score of the treated group clearly decreased, while that of the placebo group displayed nearly no change.

### 3.6. Bone Marrow Edema (BME)

BME-like lesions are also associated with the pathogenesis of osteoarthritis and are characterized by histologic abnormalities such as bone marrow necrosis and fibrosis, in addition to trabecular abnormalities [55]. Therefore, MRI was also used to assess BME before and after 12 and 24 months of treatment (Figure 3D). Before the sham treatment, the length of BME in the placebo group was 1.9 ± 0.74 mm; an increase in BME length was observed at 12 and 24 months (2.0 ± 0.53 mm and 2.1 ± 0.64 mm, respectively *p* < 0.05). Interestingly, compared to the placebo, the BME length before SVF treatment (2.4 ± 0.34 mm) was significantly larger than after 12 and 24 months of treatment (1.5 ± 0.5 mm and 0.9 ± 0.73 mm, respectively (*p* < 0.05). On the whole, these results indicate a reduction in the formation of BME-like lesions after SVF treatment. 

### 3.7. Comparative Assessment of the VAS Score between KL2 and KL3 Groups

**Figure 4:**
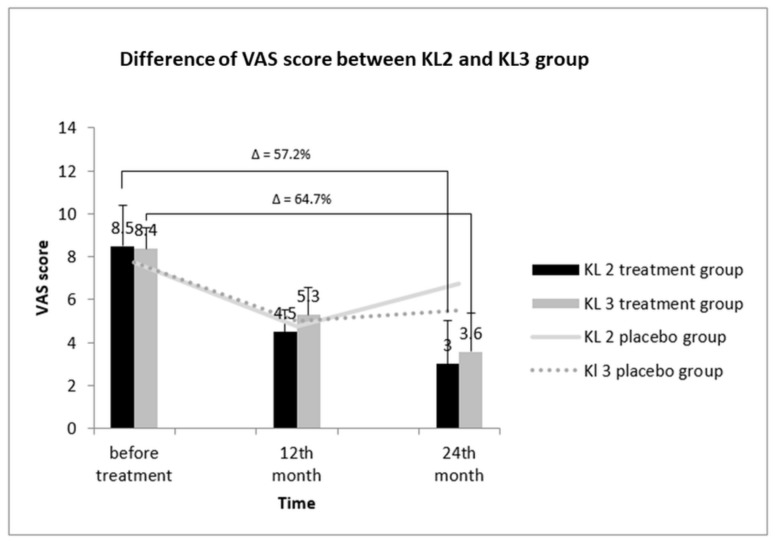
VAS scores of KL-grade 2 and 3 patients in SVF-treated OA groups at 12 and 24 months. After treatment, improvement was noted in patients with KL grade 2 and KL grade 3 (64.7% and 57.2%). Δ: percentage of reduction in VAS score.

The X-ray image-derived KL grading scale is a gold standard for determining the severity of OA, on the basis of which, the total OA patients were divided into KL2 and KL3 groups [6]. Further, we analyzed the relation between KL grading and VAS score in KL2 and KL3 treatment groups (Figure 4). Before treatment, the VAS score of the KL2 treatment group was 8.50 ± 1.92; it decreased to 4.50 ± 1 after 12 months. Notably, this score further decined to 3.00 ± 2 after 24 months of treatment, indicating a 57.2% decrease in the VAS score. Next, the effect of the placebo on VAS score of KL2 group was assessed. We found no considerable reduction in the VAS score of the KL2 placebo group before and after 24 months of placebo administration. Similarly, a reduction in the VAS score of the KL3 group was also observed post-treatment. Before treatment, the VAS score was 8.36 ± 1.00 and was reduced after 12 and 24 months of treatment to 5.29 ± 1.27 and 3.57 ± 1.79, respectively. This reduction in the VAS score was 64.7% after 24 months compared to the value before treatment. Taken together, the improvement in the pain status of KL3-grade patients was better than for KL2-grade patients.

### 3.8. Correlation between WOMAC Score and KL Grades to Determine Treatment Efficacy

**Figure 5:**
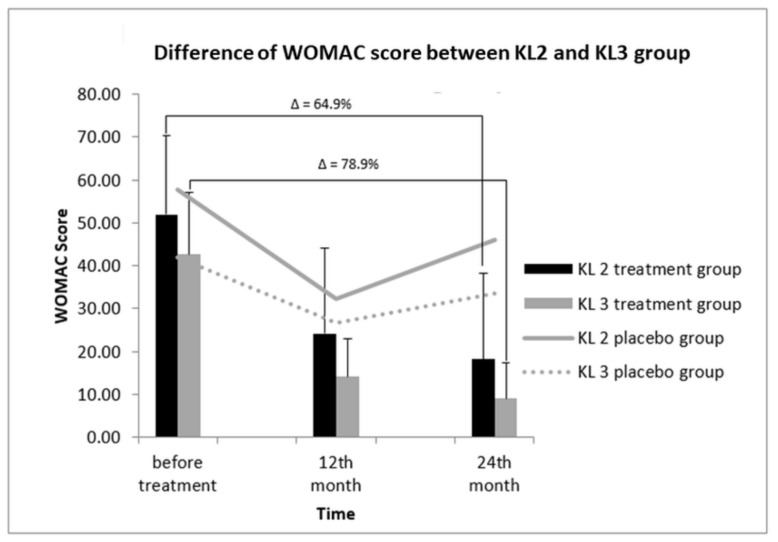
WOMAC scores in KL-grade 2 and 3 patients after SVF therapy at 12 and 24 months. After treatment, the reduction of the WOMAC score in KL-grade 3 patients was comparatively greater than that observed in KL-grade 2 patients (78.9% vs. 68.9%). The WOMAC scores of KL-grade 2 and 3 patients in the placebo group remained constant. Δ: percentage of reduction in WOMAC score.

Similarly, after treatment of KL2- and KL3-grade patients, differences in the WOMAC scores between the two groups were observed (Figure 5). The WOMAC scores before treatment in KL2 and KL3 patients were 52.00 ± 18.26 and 42.64 ± 14.51, respectively. After 12 and 24 months of treatment, the WOMAC score of the KL2 treatment group revealed a decreasing pattern, being 24.25 ± 19.77 and 18.25 ± 20.07, respectively. Similarly, the WOMAC score of the KL3 treatment group also dropped after 12 and 24 months of treatment to 18.21 ± 8.20 and 9.00 ± 8.46, respectively; however, this decline found to be not significant. Overall, compared with the value before treatment, at 24 months, the percentage of WOMAC score of the KL3 group was reduced with respect to that of the KL2 group (78.9% vs. 64.9%), indicating a greater extent of improvement in the KL3 group.

### 3.9. Relative KL Grading and Lysholm Score between KL2 and KL3 OA Groups

**Figure 6:**
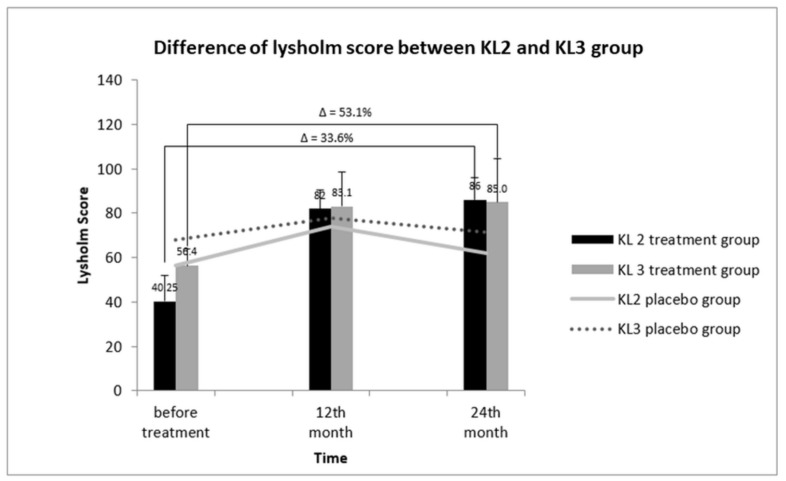
Lysholm scores of KL-grade 2 and 3 patients after SVF therapy at 12 and 24 months. After 24 months of treatment. The increase of the Lysholm score in KL-grade 3 patients was comparatively greater than that in KL-grade 2 patients (33.6%. vs. 53.1%). Δ: percentage of improvement in lysholm score.

The impact of KL-OA grades on the Lysholm score is represented in Figure 6. Before treatment, the Lysholm score of the KL2 treatment group was 40.25 ± 11.18; it increased rapidly to 82 ± 9.38 after 12 months of treatment. However, after 24 months, only a marginal increase in the Lysholm score in the KL 2-treated group to 86 ± 10.42 was observed, corresponding to a 33.6% increase compared to the value before treatment (40.25 ± 11.18 vs 86 ± 10.42). The Lysholm score of the KL3 treatment group followed almost a similar pattern as that of the KL2 group. The score before treatment was 56.4 ± 11.66 and increased to 83.1 ± 8.52 after 12 months of treatment, showing an increase of 53.1%. However, a slight increase to 85.0 ± 10.19 after 24 months of treatment was observed. These data showed that the improvement of the KL3 group were greater than that the KL2 group.

### 3.10. Comparative Outerbridge Score (OS) between KL2 and KL3 Groups

**Figure 7:**
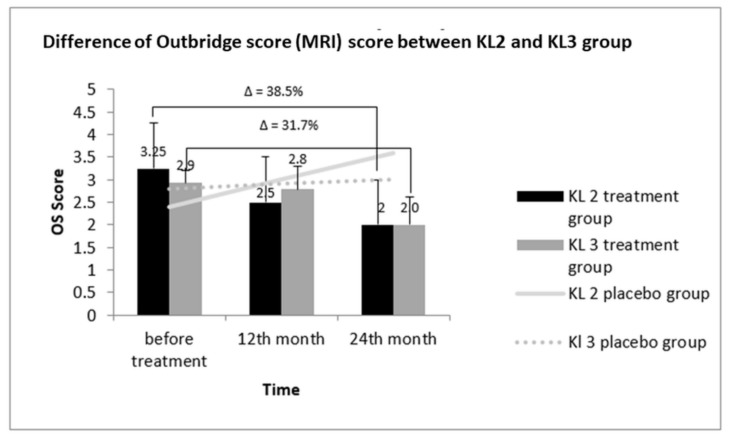
OS in SVF-treated and placebo groups of KL-grade 2 and 3 patients after at 12 and 24 months. After treatment, improvement was noted in KL-grade 2 and KL-grade 3 patients (38.5% and 31.7%). Δ: percentage of reduction in OS score.

The comparative profile of cartilage injury, as measured by the OS score in KL2 and KL3 patients after treatment, is represented in Figure 7. No significant improvement was observed in the OS of the KL 2 placebo group up to 24 months of treatment when compared to the scores before treatment. Specifically, the OS of the KL2 treatment group before treatment was 3.25 ± 0.55; however, it decreased to 2.58 ± 0.70 after 12 months of treatment and further reduced to 2.0 ± 1.19 after 24 months. The net decrease in OS score after 24 months of treatment was 38.5%. In accordance with the OS score pattern of the KL2 treatment group, the OS score of the KL3 treated group also decreased after 12 and 24 months of treatment to 2.8 ± 0.51 and 2.0 ± 0.61, respectively, compared to the value before treatment of 2.9 ± 0.51. The OS score of the KL3 placebo group showed a linear increase after 24 months of treatment. In contrast to the WOMAC and VAS scores, OS showed no difference in improvement between KL2 and KL3 groups.

## 4. Discussion

SVF contains a heterogeneous cell population of progenitor cells and ADSCs, which possess enhanced therapeutic potential against immune disorders, degenerative tissue pathologies, and other ischaemic conditions [37]. The complexity of knee OA related to pain, stiffness, muscle atrophy, and ligament damage has made its treatment difficult. Surgical procedures and drugs for controlling pain and inflammation have proven to be inadequate [56]. However, recent developments in regenerative therapy have provided the opportunity to address the bottlenecks associated with OA treatment. Similar to other MSCs, SVFs containing ADSCs are considered a better candidate at par with ADSCs and in some case better than pure ADSCs [35,36]. Therefore, this study assessed the efficacy of SVF treatment in OA therapy. In particular, the VAS, WOMAC, Lysholm, and MRI-based Outerbridge scores were evaluated to assess the improvement in OA status. VAS, WOMAC, and Lysholm score, closely represent the real-time status of OA; therefore, they are precise enough to evaluate the effectiveness of OA treatments [57]. The VAS score is directly measured through questionnaires [58]. The level of pain is established between two extreme points—no pain at all and worst pain imaginable [59]. This scale is simple, reliable, and valid to represent the level of pain [60]. As compared to the placebo group, a considerable reduction in the VAS score of the treatment group was observed after 24 months of treatment, reflecting an improvement of pain. On the contrary, no significant difference between the VAS scores of SVF and placebo groups after 12 months of treatment was found when an arthroscopic procedure was conducted prior to SVF administration. During this process, the inflamed tissues in both the groups were removed, which might have suppressed the pain symptom even in the placebo group, compared to the pain level before treatment. In coherence to our study, the SVF/PRP treatment has also been reported to improve the VAS score of OA patients [58]. A recent clinical study approved by the Japanese Regenerative Medicine Safety Act has documented a 40% decrease in VAS score after SVF treatment [61]. Furthermore, our study demonstrated that the WOMAC score was considerably decreased after 24 months of SVF treatment. These decreases in VAS (Figure 2A) and WOMAC scores (Figure 2B) compared to placebo groups were significant, which indicates improvement in the painful condition of OA patients. Following the pattern of VAS and WOMAC scores, the Lysholm score was also employed to assess the improvement in quality of life and status of instability post-surgery and post-treatment. The current modified Lysholm score is based on eight features, including limp, support, locking, instability, pain, swelling, stair climbing, and squatting [62]. Lysholm is mainly based on the opinion of a patient assessing function and stability of treatment; an increased score indicates improved quality of life. Our study indicates a significant effect of SVF on the Lysholm score in OA patients 24 months post-treatment as compared to the placebo group. This increase in Lysholm score is an indication of patient relief to therapy. This result is in accordance with previous studies carried out to assess the efficacy of SVF therapy in OA treatment.

Further, the level of cartilage injury was assessed on the MRI-based OS score. An increase in OS score represents a loss of cartilage thickness. In this study, initially there was no significant difference between the OS scores of the treatment and placebo groups; however, a significant decrease in OS score was observed in the treatment group compared to the placebo group after 24 months of treatment (*p* < 0.05). These data establish the role of SVF in improving the BME score which is used as an indicator of knee OA progression and is characterized by increased accumulation of fluid [63]. A significant decrease in the BME score was observed in the SVF-treated group after 24 months of treatment with respect to the placebo which showed increased tendency. The comparison of the BME and OS scores of placebo and treatment groups at the end of 24 months of treatment indicated considerable improvements in the cartilage phenotype, particularly increased thickness. 

KL classification is a five-grade scaling system in which the radiographs of eight joints are used to grade knee OA [64]. In this study, KL2- and KL3-grade patients were included to assess the effect of SVF treatment on the OA grade. On the basis of the decrease in WOMAC score and the increase in Lysholm score and considering the static response of the placebo groups during the 24 months of this study, it can be inferred that the SVF treatment was more effective in KL3-grade patients than in KL2-grade patients. The greater improvement of KL3-grade group patients might be attributed to the subjective assessment of the VAS score, WOMAC score, and Lysholm score, whereby patients with a severe condition tend to feel a greater improvement. In contrast, in the case of MRI scores (OS and BME scores) which are based on objective assessment, no differences between the two groups were witnessed. Inflammation plays a central role in pathogenesis of osteoarthritis and significantly contributes to joint pain [65]. Hence, the reduction of pain observed by us is likely to be related to the anti-inflammatory properties of SVF cells. As a corollary, it is also plausible that the better results obtained for KL3 patients, characterized by a higher level of inflammation before treatment compared to KL2 patients, depend on a better and more profitable exploitation of the anti-inflammatory activity of SVF. On the other hand, the degenerative properties of SVF will have the same effect on KL2 and KL3 patients. 

The claim of SVF potential in improving clinical scores of OA patients might be attributed to SVF, which is a mixture of ADSCs, endothelial precursor cells (EPCs), endothelial cells (ECs), macrophages, smooth muscle cells, lymphocytes, pericytes, and pre-adipocytes [37,66]. The improvements in the clinical scores might be attributed to immuno-modulator and anti-inflammatory effects of SVF cells, which can lead to tissue remodeling. SVF cells secrete immunosuppressive and anti-inflammatory molecules like IL-10, IL-1, receptor antagonist (IL-1ra), indoleamine 2,3-dioxygenase, transforming growth factor (TGF)-β, and prostaglandin [67]. Further, the anti-fibrotic effect of ASDC might also play a role through the secretion of HGF or adrenomodullin, thereby reducing the fibrotic activity of overexpressed TGF-β1 and its target genes, such as collagen type I, type III, and α-SMA in OA knee [68,69,70]. 

Besides these therapeutic activities, the regenerative ability of SVF may be due to ADSCs differentiation potential into chondrocytic and osteocytic cells lineages. EPCs may also induce angiogenesis by releasing growth factors such as vascular endothelial growth factor (VEGF) and insulin-like growth factor-1 (IGF-1) [71]. Macrophages and monocytes have been demonstrated to mediate the immune response through secretion of various cytokines [72]. These macrophages are modulated by T regulatory cells, which may possess immunosuppressive characteristics [73]. In a mouse model, the pericytes found in SVF were able to regenerate the muscle tissue [74], which indicates their therapeutic potential role in knee joint. Eventually, stromal cells can secrete extracellular matrix components which improve cellular adhesion, migration, cell–matrix interactions, and regeneration [75,76]. To our knowledge, this is the first study reporting time- and KL grade-dependent changes of intra-articularly transplanted SVF in osteoarthritic patients over a period of two years. The main limitation of this study is the small sample size. However, even a small sample might have some valid scientific merit with cost effectiveness [77,78]: on the basis of it we have inferred SVF-mediated therapeutic clinical outcomes in this study. To overcome this limitation, this study will be extended to a larger population and conducted for a longer time.

## 5. Conclusions

On the basis of the improvements observed in treated patients during follow-up and the behavior of the placebo group, our study revealed a trend toward a better efficacy of SVF with the microfracture method for OA treatment over a period of two years. We also inferred that the SVF therapy is more effective in KL 3-grade OA patients compared with KL 2-grade OA patients.

## Figures and Tables

**Table 1:** Population characteristics of the patients. BMI: Body mass index.

Characteristics	Placebo Group	SVF-Treated Group
Age	58.2 ± 5.70	59 ± 6.04
Sex		
Male	3	5
Female	12	13
BMI		
Normal: Overweight: Obese	9:5:3	11:5:3
KL grades		
KL2	5	4
KL3	10	14
